# A multifunctional thienothiophene member: 4-thieno[3,2-*b*]thiophen-3-ylbenzonitrile (4-CNPhTT)

**DOI:** 10.55730/1300-0527.3608

**Published:** 2023-09-30

**Authors:** Recep İŞÇİ, Turan ÖZTÜRK

**Affiliations:** 1 Department of Chemistry, İstanbul Technical University, İstanbul, Turkiye; 2 TÜBİTAK UME, Chemistry Group Laboratories, Kocaeli, Turkiye

**Keywords:** Thienothiophene, conjugated material, electropolymerization

## Abstract

Thieno[3,2-*b*]thiophene (TT) has been attracting significant attention in the field of organic electronics and optoelectronics. In this study, a useful building block of TT derivative 4-thieno[3,2-*b*]thiophen-3-ylbenzonitrile (**4-CNPhTT)**, developed by our group and possessing a strong electron-withdrawing 4-CNPh moiety, is reviewed as it has been the source of the development of various organic electronic materials. Some optic and electronic properties are discussed based on electrochemical polymerization of **4-CNPhTT** performed using cyclic voltammetry, and spectroelectrochemical measurements are conducted to investigate the optical variations of the polymer film upon doping. Moreover, **4-CNPhTT** is clarified by scanning electron microscopy at different magnitudes ranging from 100 to 500 μm, supported by the single X-ray crystal structure. The thermal properties of **4-CNPhTT** are investigated by thermal gravimetric and differential thermal analyses. All of the observed properties demonstrate that **4-CNPhTT** has the potential of shedding light on the development of new materials for electronic and optoelectronic applications within the TT family.

## 1. Introduction

Organic conjugated materials, including both polymers and small molecules, have emerged as versatile materials to be utilized in electronic-optic [1], sensing [2], security [3], healthcare [4,5], and renewable and sustainable energy [6] applications. Light weight, flexibility, chemical stability, low cost, and adaptability to different shapes are the main factors playing significant roles in making conjugated materials an integral part of our daily lives [7–10]. Conjugated materials have been used quite frequently, particularly in the development of energy and energy-based applications, such as organic light-emitting diodes (OLEDs), organic field-effect transistors, capacitors, sensors, and solar cells. In all of these applications, thienothiophenes (TTs) are of great interest in material chemistry as parts of both polymeric and small molecules [11–13].

**Figure 1:**
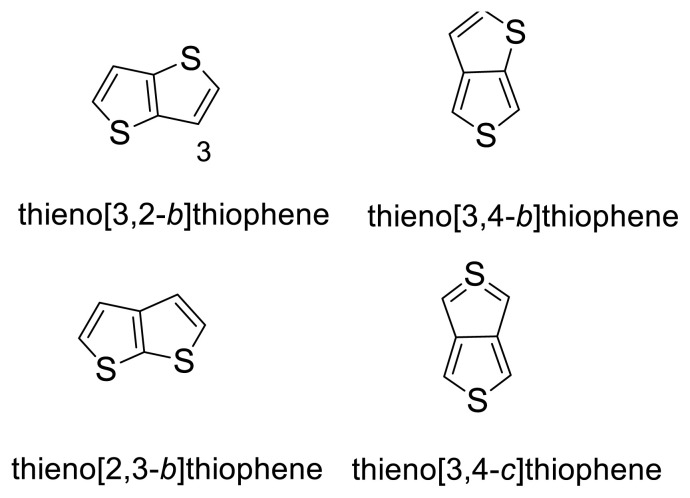
Thienothiophene (TT) isomers.

TTs, having the structure of two fused thiophenes, are electron-rich organic compounds. They have four structural isomers, thieno[3,2-*b*]thiophene, thieno[3,4-*b*]thiophene, thieno[2,3-*b*]thiophene, and thieno[3,4-*c*]thiophene, among which the most widely used form is thieno[3,2-*b*]thiophene (Figure 1) [14–16]. This is mainly due to better extended conjugation, planar ring structure, extended p-conjugation, chemical stability (except thieno[3,4-*b*]thiophene), and property of creating intermolecular S---S interactions. These properties make TTs appealing building blocks for the designing and synthesizing of semiconducting materials with improved air stability and good transport performance [17,18].

**Scheme 1:**
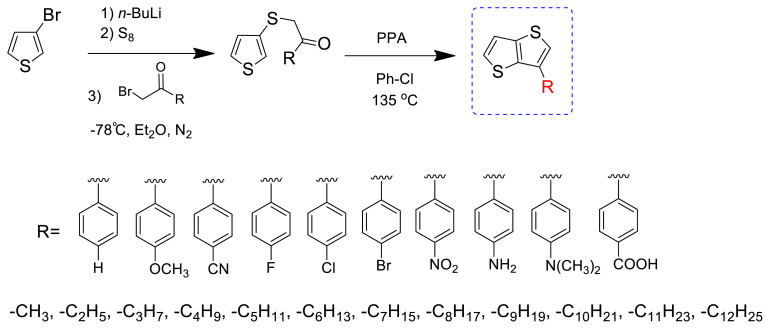
Functionalized TTs synthesized in two steps.

The versatile properties of TTs have led various research groups to develop functionalized TTs as conjugated organic materials. Our group is one of the leading groups exploring the functionalization, photophysical properties, and applications of TTs [19–23]. A simple synthetic strategy developed by Öztürk’s group has shortened the cumbersome four-step synthetic method reported in the literature to two steps with high yields, which has particularly led to the introduction of various aryl and alkyl groups to the 3-position of thieno[3,2-*b*]thiophene (Scheme 1) [24–28]. This method has now been used by some groups and declared as a convenient method, with the synthesis being performed via alternative synthetic routes, simplifying the variation of the side chains of the outer thiophene site with a universal TT building block that can be produced with good yields from inexpensive starting materials [29–31]. Such substitutions caused remarkable changes in the electronic and optical properties of polymeric and small molecules constructed using TTs as building blocks [32–39].

**Figure 2:**
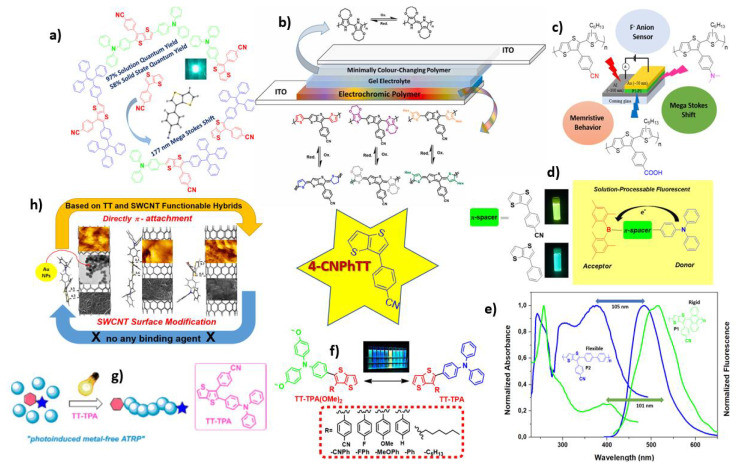
Remarkable examples of **4-CNPhTT** derivatives used in material science.

As **4-CNPhTT** has been a very productive building block for the preparation of various organic materials, its applications are summarized in Figure 2. Some notable examples are as follows: (i) 58% solid-state and 98% solution (THF) quantum yields of the material prepared by Suzuki coupling reaction with triphenylamine (TPA) and its OLED application (Figure 2a) [23]; (ii) polymers with thiophene, EDOT, and alkyl thiophene and their electrochromic devices (Figure 2b) [33]; (iii) polymerization with 3-hexylthiophene, fabrication as a memory device, and use of the polymer as a fluorine sensor (Figure 2c) [26]; (iv) preparation of donor-p-acceptor type solution processible OLED through the combination of TPA and dimesityl boron (DMB) (Figure 2d) [24]; (v) polymers with biphenyl and anthracene with a comparative study of their molecular orientations (Figure 2e) [22]; (vi) synthesis and properties of TPA/4,4′-dimethoxytriphenylamine (TPA(OMe)_2_)-functionalized fluorophores having mega Stokes shifts of up to 179 nm, optical band gaps ranging from 2.86 to 3.08 eV, and fluorescence lifetimes from 2.05 to 4.70 ns (Figure 2f) [21]; (vii) use as a photoactivator and deactivator for the preparation of polymers, applying the photoinduced metal-free atom transfer radical polymerization method (Figure 2g) [34]; and (viii) the first example of noncovalent functionalization of sidewalls of single-wall carbon nanotubes (SWCNTs) without requiring any binding agents (TT-based SWCNT hybrids) (Figure 2h) [25].

In this study, along with an overview of the applications of the compounds possessing **4-CNPhTT**, some properties for the photophysical, spectroelectrochemistry, electropolymer, thermal, and surface characterization of core unit **4-CNPhTT** without any substituents are reported to emphasize its importance as a building block in organic material chemistry.

## 2. Materials and methods

All reagents were purchased from Aldrich and Acros and used without further purification. All of the solvents used in the syntheses were of technical grade and freshly distilled prior to use. The solvents used in the spectroscopic measurements were of spectroscopic grade. Flash chromatography was performed with silica gel of ≤0.063 μm. Fluorescence spectra were recorded on a HITACHI F-4500 fluorescence spectrophotometer. UV-Vis measurements were recorded on a HITACHI U-0080D spectrophotometer. Cyclic voltammetry and spectroelectrochemistry were examined with a Princeton Applied Research Versa STAT 3. Scanning electron microscopy (SEM) images were recorded using a HITACHI SU 500 FEG-SEM instrument. Images were obtained at acceleration voltage of 2.0 kV in high vacuum.

## 3. Results and discussion

**Scheme 2:**
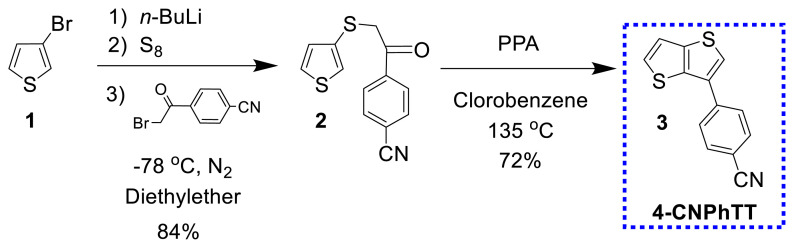
Synthesis of **4-CNPhTT**.

The design and synthesis of the core unit, 4-thieno[3,2-*b*]thiophen-3-ylbenzonitrile (**4-CNPhTT**) **3**, was conducted starting from 3-bromothiophene (**1**). Monoketone 4-[2-(thiophen-3-ylsulfanyl)acetyl]benzonitrile **2** was constructed in a one-pot, three-step reaction with 84% yield including (i) lithiation of 3-bromothiophene with *n*-butyllithium at −78 °C and (ii) additions of elemental sulfur and (iii) 4-(2-bromoacetyl)benzonitrile. The ring closure reaction of **2** in the presence of polyphosphoric acid in refluxing chlorobenzene gave **3** (**4-CNPhTT**) in 72% yield (Scheme 2) [21–23].

**Figure 3:**
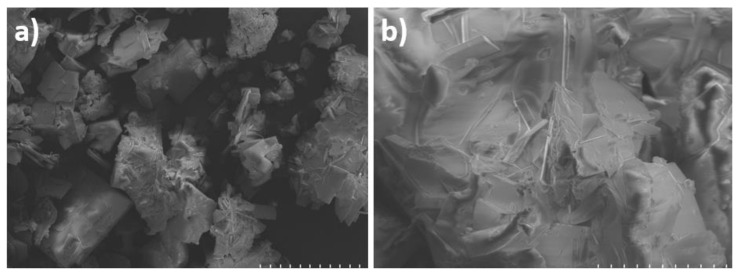
SEM images of **4-CNPhTT** in powder form at **a)** low magnification and **b)** high magnification and acceleration voltage of 2.0 kV using a HITACHI SU 500 FEG-SEM instrument.

**Figure 4:**
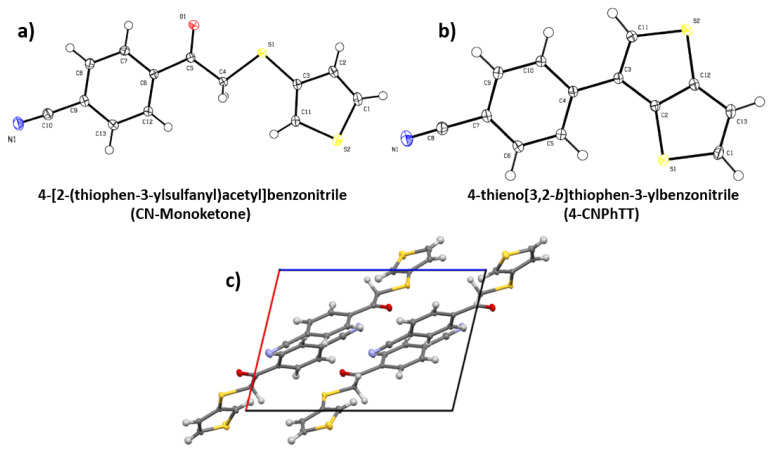
Molecular drawings of **a)** the monoketone and **b) 4-CNPhTT** (**3**) (thermal ellipsoids drawn at 50% probability) and **c)** p–p stacking interaction of monoketone **2**.

The surface morphology of **4-CNPhTT** was investigated using SEM. Considering that the SEM analysis was conducted on a very small area, several images were recorded from different parts at low and high magnifications, which indicated a consistent crystal morphology of micrometer size (Figure 3). The reason for this crystal fragmentary morphology was the strong p–p stacking interactions of the CNPh moiety. Moreover, single crystal structures in a monoclinic lattice of the monoketone and **4-CNPhTT** [23] and the cubic packing conformation of the monoketone, which demonstrated strong p-p stacking interaction between CNPh units, were consistent with the crystalline appearance in SEM images of **4-CNPhTT** (Figure 4).

**Figure 5:**
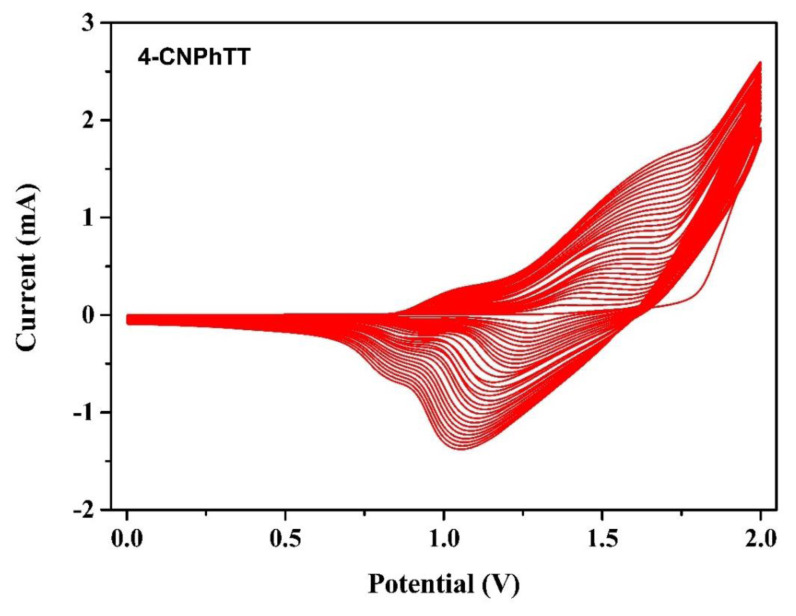
Electropolymerization of **4-CNPhTT**.

Electropolymerization of **4-CNPhTT** was studied in a standard three-electrode cell using silver wire as a pseudo-reference electrode, platinum wire as a counter electrode, and an ITO-coated glass slide as a working electrode. Electropolymerization of the monomers (1 × 10^−3^ M) was performed in an anhydrous solvent mixture (ACN/DCM, 90/10 v/v) in the presence of NaClO_4_ (0.1 M)/LiClO_4_ (0.1 M) as a supporting electrolyte at a scan rate of 100 mV s^−1^ under ambient conditions. A multiple cyclic voltammogram of the repeated scanning electropolymerization of **4-CNPhTT** is depicted in Figure 5. Its polymerization was observed by means of deposition of the polymers on the Pt electrode surface and as an increase of oxidation and reduction peaks. While the oxidation potential of monomer **4-CNPhTT** started at 1.40 V [23], the oxidation wave for **p(4-CNPhTT)** shifted to 0.84 V, which was considered as evidence of successful electropolymerization.

**Figure 6:**
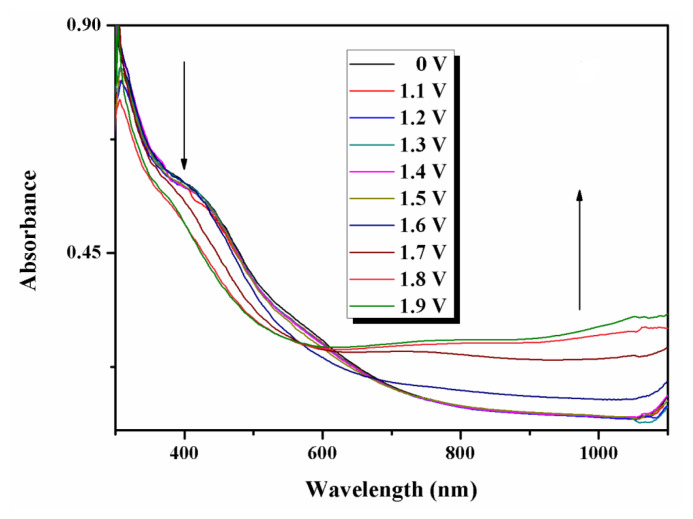
UV-Vis absorption spectra of **p(4-CNPhTT)** film obtained at different applied potentials in the range of 0.0–1.9 V.

A better understanding of the nature of a redox process could be achieved through the analysis of UV-Vis spectra registered for increasing potential. Spectroelectrochemistry of **4-CNPhTT** was thus investigated (Figure 6). The polymer-coated glasses were added to UV cuvettes as working electrodes, filled with monomer-free electrolyte solution. Pt and Ag wires were used as counter and reference electrodes, respectively. Regarding the polymer, **p(4-CNPhTT)**, changes in its absorbance were measured in situ as a function of potential change starting from 0 V, which was gradually increased up to 1.9 V. The alterations of the absorbances were observed through stepwise UV-Vis measurements. In the overall appearance of the polymer, as a result of electrochemical doping, the absorption peaks in the neutral state decreased in intensity, while new polaron and bipolaron peaks (interband states) formed around 1000 nm, which is the characteristic behavior of conducting polymers.

**Figure 7:**
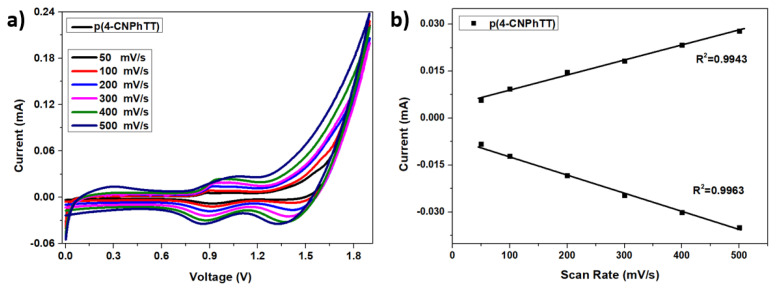
**a)** CVs of **p(4-CNPhTT)** films obtained at different scan rates in ACN containing 0.1 M Bu_4_NPF_6_. **b)** Plot of anodic and cathodic current peaks as a function of scan rate.

Cyclic voltammograms and the scan-rate dependence of the current densities of the polymer were recorded at different scan rates (50–500 mV s^−1^) in the 0.1 M Bu_4_NPF_6_/ACN electrolyte system to investigate its electrochemical properties (Figure 7). Linear correlation between the peak current and scan rate indicated a well-coated film on the electrode surface. The electrochemical process was found to be diffusion-controlled and reversible.

**Figure 8:**
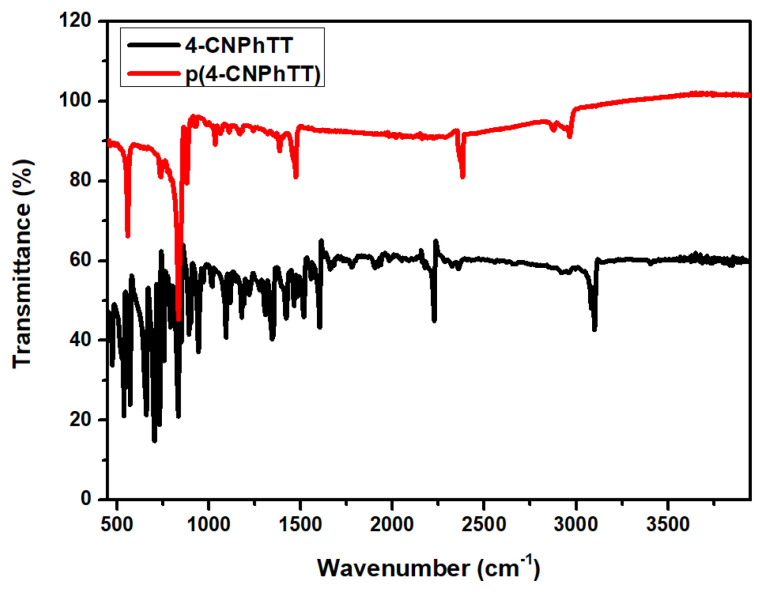
FT-IR spectra of **4-CNPhTT** powder and the **p(4-CNPhTT)** film.

**4-CNPhTT** and the polymer film were characterized by FT-IR spectroscopy (Figure 8), where the peaks at 750 and 1100 cm^−1^ were interpreted to be C–H bending vibrations of the TT moiety. The electropolymerization resulted in the disappearance of these peaks due to the coupling that took place through these side positions.

**Figure 9:**
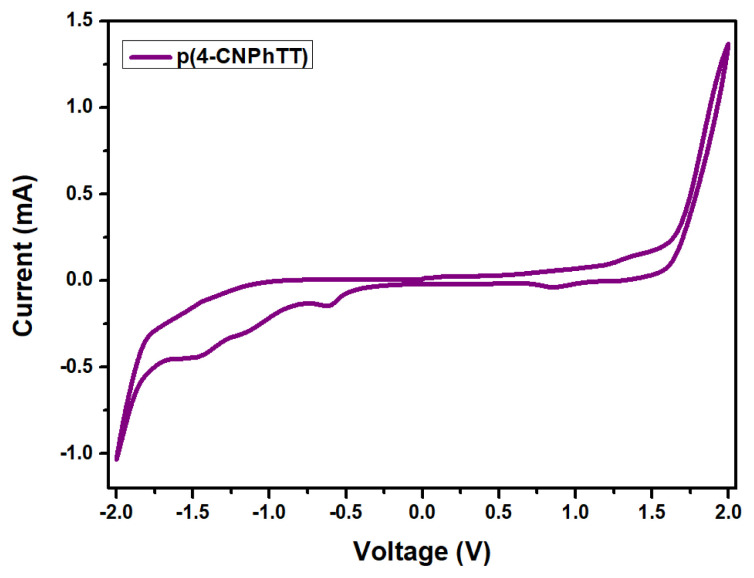
CV of **p(4-CNPhTT)**.

The electrochemical behavior of the polymer was investigated by cyclic voltammetry in an acetonitrile mixture in the presence of tetrabutylammonium hexafluorophosphate (Bu_4_NPF_6_, 0.1 M) as a supporting electrolyte at a scan rate of 100 mV s^−1^ (Figure 9). Based on the onset potentials, the HOMO/LUMO energies of **p(4-CNPhTT)** were calculated to be −5.61/−3.58, using the equations of [HOMO = – (4.4 + E_ox,onset_)] and [LUMO = – (4.4 + E_red,onset_)]. The electronic band gap of the polymer was then calculated as 2.03 eV using the equation E_CV_ = |HOMO – LUMO|.

**Figure 10:**
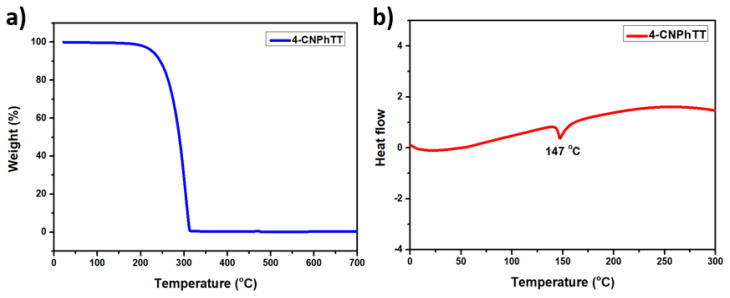
**a)** TGA and **b)** DTA graphs of **4-CNPhTT**.

The thermal properties of **4-CNPhTT** were studied by applying thermal gravimetric analysis (TGA) up to 700 °C at a heating rate of 10 °C min^−1^ under N_2_ atmosphere (Figure 10a). The TGA curve showed detailed decomposition at 313 °C. The purity of the compound was further confirmed with a sharp endothermic peak observed in the differential thermal analysis curve at 147 °C, which matched the melting point of **4-CNPhTT** (Figure 10b).

## 4. Conclusion

**4-CNPhTT**, 4-thieno[3,2-*b*]thiophen-3-ylbenzonitrile, has been the source of the development of many organic materials as a multifunctional TT member. In this work, this main core, **4-CNPhTT**, was examined in terms of electronic, spectroscopic, surface, and thermal analysis along with a short review to emphasize its usefulness as a building block for various organic electronic and optoelectronic materials. **4-CNPhTT** was electropolymerized by repeated scanning method to obtain **p(4-CNPhTT)**, and its spectroelectrochemical properties were investigated by changing the potentials between 0.0 V and 1.9 V. Furthermore, the SEM properties of **4-CNPhTT** were measured at different magnitudes and the results were found to be in good agreement with the X-ray crystal structure. This study focused on the properties of the **4-CNPhTT** unit as a main core of various organic materials and reported its features in detail. It seems that **4-CNPhTT** will continue to contribute to the progress of many different applications in the area of organic semiconductors.
